# Pterostilbine, an active component of blueberries, sensitizes colon cancer cells to 5-fluorouracil cytotoxicity

**DOI:** 10.1038/srep15239

**Published:** 2015-10-16

**Authors:** Mai F. Tolba, Sherif Z. Abdel-Rahman

**Affiliations:** 1 Department of Pharmacology and Toxicology, Faculty of Pharmacy, Ain Shams University, Cairo 11566, Egypt; 2 Biology Department, School of Sciences and Engineering, The American University in Cairo, New Cairo, Egypt; 3 Department of Obstetrics and Gynecology, The University of Texas, Medical Branch, Galveston 77555, USA

## Abstract

Although colorectal cancer (CRC) treatment with 5-fluorouracil (5-FU) is the first line of therapy for this debilitating disease, treatment effectiveness is often hampered by the development of drug resistance and toxicity at high doses. ER-β can play an important role in CRC development and possibly in its response to therapy. Pterostilbene (PT) possesses antioxidant and anticancer effects that are mediated by ER-β. In the current study, we test the hypothesis that PT sensitizes colon cancer cells to 5-FU and we examine the underlying mechanism(s) by which PT exerts its cytotoxic effects in CRC cells. Our data indicate that PT exhibited a more potent cytotoxic effect in Caco-2 compared to HCT-116 cells. PT/5-FU co-treatment was more effective in Caco-2 cells. Our data indicate that ER-β is expressed at higher levels in Caco-2 cells and its levels are further boosted with PT treatment. PT significantly suppressed Akt and ERK phosphorylations, and enhanced FOXO-1 and p27^kip1^ levels in Caco-2 cells. PT also induced a significant increase in Caco-2 cells at pre-G phase coupled with increased Bax/Bcl-2 ratio and PARP cleavage. These results provide a rationale for novel combination treatment strategies, especially for patients with 5-FU-resistant tumors expressing ER-β protein.

Colorectal cancer (CRC) is one of the most commonly diagnosed solid tumors worldwide. It is ranked as the second cause of cancer-related death in males and the third cause of cancer-death in females in developed countries^1^. The chemotherapeutic agent 5-fluorouracil (5-FU) is the first line of therapy for this debilitating disease. Treatment with 5-FU represses the growth of cancer cells by acting as a false substrate to thymidylate synthase enzyme that incorporates its metabolites into DNA and RNA leading to defective synthesis and subsequent induction of apoptosis. However, treatment effectiveness is hampered by resistance to therapy and toxicity that develops at high doses^2^. Estrogen receptor(ER) status is suggested to be implicated in the pathogenesis of CRC. The ER-β is the predominant ER in the colorectal epithelium and studies indicated that ER-β is expressed at higher levels in normal colon mucosa compared to adenomatous polyps. Importantly, ER-β expression is significantly reduced in CRC compared with normal colon tissue^3^. The expression of ER-β is directly correlated with apoptosis and inversely correlated with cell proliferation^4^. Treatment of MC38 colon cancer cell line with diaryl-propionitrile, which acts as ER-β agonist, reduced cell proliferation rate^5^. Likewise, transfection of ER-β into SW480 colon cancer cells suppressed cell proliferation^3^. ER-β is associated with stage and grade of the disease, and an inverse relationship between ER-β expression and tumor progression has been reported in cell lines and clinical samples^3^,^6^,^7^. As such, it is hypothesized that estrogen-mediated signaling exerts a protective role in CRC and its modulation could provide another therapeutic option for the disease^8^.

Stilbenes, including resveratrol and pterostilbene (PT), are a class of naturally occurring phenolic compounds that exhibit a wide spectrum of biological functions including anticancer activity^9^,^10^,^11^. Berries are considered a rich source for PT and its abundance varies between different types of berries. Some varieties of blueberries contain up to 15 μg PT per 100 gm (1 cup) of berries^12^. PT is a structural analogue to resveratrol and is characterized by the presence of 2 methoxy groups instead of the hydroxyl groups of resveratrol^13^. PT was reported to be superior to resveratrol in suppressing the formation of aberrant foci in a mouse model of azoxymethane-induced colon carcinogenesis^14^. Moreover, PT surpasses resveratrol in its inhibition for the DNA synthesis as well as suppressing pro-inflammatory mediators (iNOS and COX-2) in colon cancer cells^15^. *In vitro* studies showed that PT possesses cytotoxic activity against CRC cells^16^,^17^ and that it is more potent compared to resveratrol in inhibiting CRC cell proliferation^18^. Furthermore, PT strongly inhibits colon cancer tumors growth in nude mice carrying human colorectal carcinoma COLO 205 tumor xenografts^17^. The growth inhibitory effects of PT were demonstrated to be through an ER-β-mediated mechanism^19^. As such, PT could constitute a promising therapeutic candidate for CRC by acting as a chemosensitizer to conventional therapy of the disease. The chemosensitizing effect of PT in CRC has not been investigated before. In the current study, we test the hypothesis that PT sensitizes colon cancer cells to 5-FU. We also examine the underlying mechanism(s) by which PT exerts its cytotoxic effects on colon cancer cells.

## Results

### Effect of PT on the cytotoxicity of 5-FU in colon cancer cells

**Figure 1:**
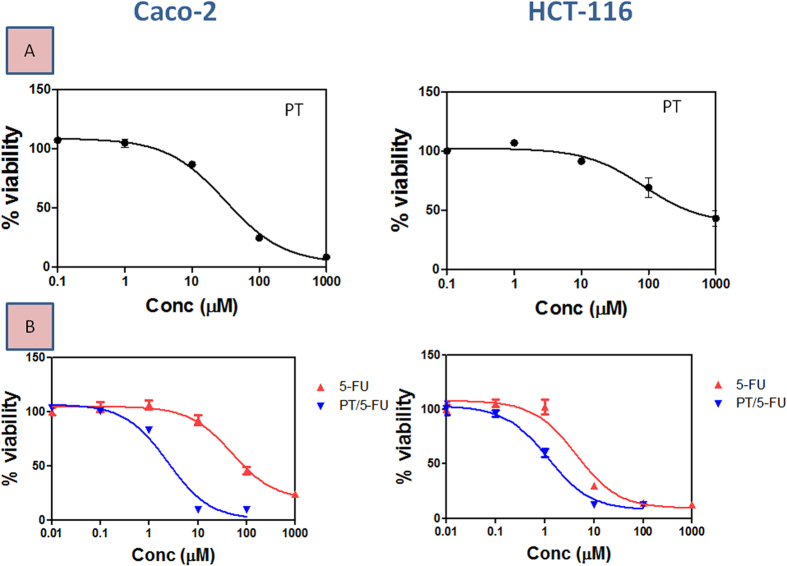
Concentration-response plots of PT (**A**), 5-FU and 5-FU in combination with PT (**B**) in Caco-2 and HCT-116 colon cancer cell lines after 72 h. Data are means ± SD (n = 3).

To investigate the effect of PT on the cytotoxicity of 5-FU, concentration- response curves of 5-FU in both Caco-2 and HCT-116 cell lines were assessed and compared to those obtained after co-treatment with PT. Treatment with PT alone produced a significant inhibition of cell viability with median inhibitory concentration (IC_50_) values of 31.2 ± 0.42 μM and 84.4 ± 1.14 μM in Caco-2 and HCT-116 cells, respectively (Fig. 1A). 5-FU exerted a concentration-dependent growth inhibition of colon cancer cells with IC_50_ value of 46.8 ± 2.5 μM and 4.3 ± 0.85 μM in Caco-2 and HCT-116 cells, respectively. Co-treatment with PT at a ratio of 10:1 of PT: 5-FU significantly reduced 5-FU IC_50_ to 2.44 ± 0.16 μM and 1.07 ± 0.01 μM in Caco-2 cells and HCT-116 cells, respectively (P < 0.05; Fig. 1B). The reduction in 5-FU IC_50_ after co-treatment with PT was more pronounced in Caco-2 cells (19- fold) compared to HCT-116 cells (4-fold). Drug interactions were analyzed by Calcusyn software and the combination index (CI) as well as dose reduction index (DRI) values for both cell lines are summarized in Tables 1 and 2.

### Effect of PT on the viability of normal cells

The normal villous cytotrophoblast placental cells (CRL-1584, ATCC, Manassas, VA) were used to evaluate the effect of PT treatment on normal cells viability. Exposure to PT at concentrations 40 and 80 μM resulted in percent cell viability of 38.2 ± 0.36 and 27.8 ± 1.54 with reductions of about 61.8% and 72.2% compared to control (Supplementary Fig. 1).

### Effect of PT on ER-β protein expression

**Figure 2:**
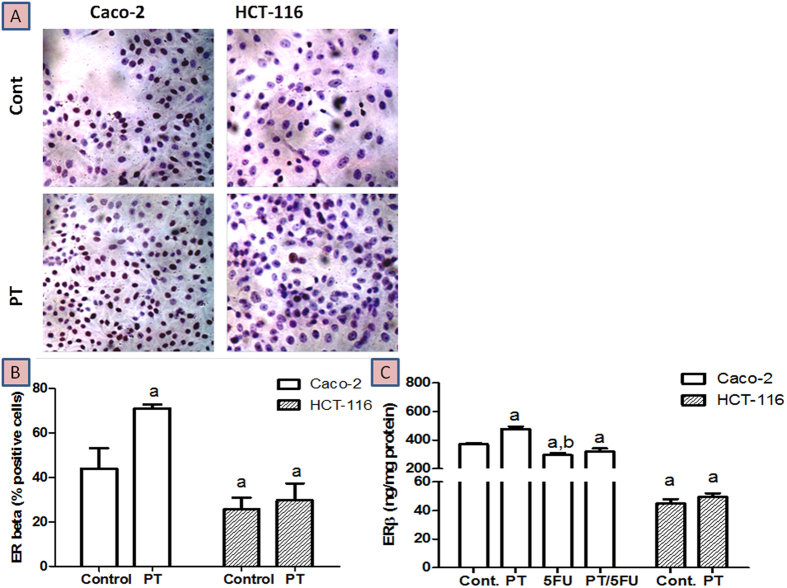
Effect of PT on the protein expression of ER-β in Caco-2 and HCT-116 colon cancer cell lines using immunocytochemistry (**A,B**) and ELISA assay (**C**).Data are means ± SD (n = 3). The experiment was done in triplicates. a indicates significant difference from Caco-2 control at p < 0.05. b indicates significant difference from Caco-2/PT group at p < 0.05.

Evaluation of ER-β status in both Caco-2 and HCT-116 cell lines using immunocytochemistry showed that ER-β abundance is higher in Caco-2 cells and that it is boosted after PT treatment (Fig. 2A,B). The results were confirmed using ER-β ELSA assay which indicated that the expression of ER-β is higher in Caco-2 cells compared to HCT-116 cells by about 8.2 fold (P < 0.001). Treatment of Caco-2 cells with PT caused significant elevation in ER-β protein expression levels by 1.29 fold (P < 0.001) compared to untreated control cells. Exposure of HCT-116 cells to PT resulted in 1.1 fold increase in ER-β expression compared to untreated cells (Fig. 2C). Furthermore, single treatment of Caco-2 with 5-FU reduced ER-β expression by 20.5% (P < 0.001). Combined treatment with PT improved 5-FU mediated suppression of ER-β by 8.9%.

### Molecular docking into ER-β active site

**Figure 3:**
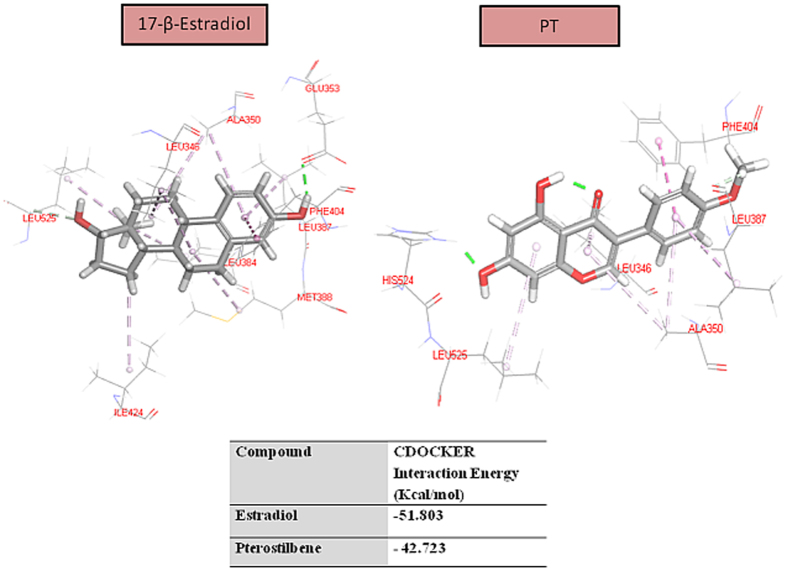
The docking poses of 17-β estradiol and PT into ER-β (1 × 7R) active site (PDB; http://www.rcsb.org/pdb).

The docking study revealed that PT docked into ER- β (1 × 7R) active site with a very high affinity value (−42.723 Kcal/mol) that is comparable to its ligand 17-β estradiol (−51.803 Kcal/mol). The docking poses for both ligands are shown in Fig. 3.

### Effect of PT on Akt phosphorylation/activation

**Figure 4:**
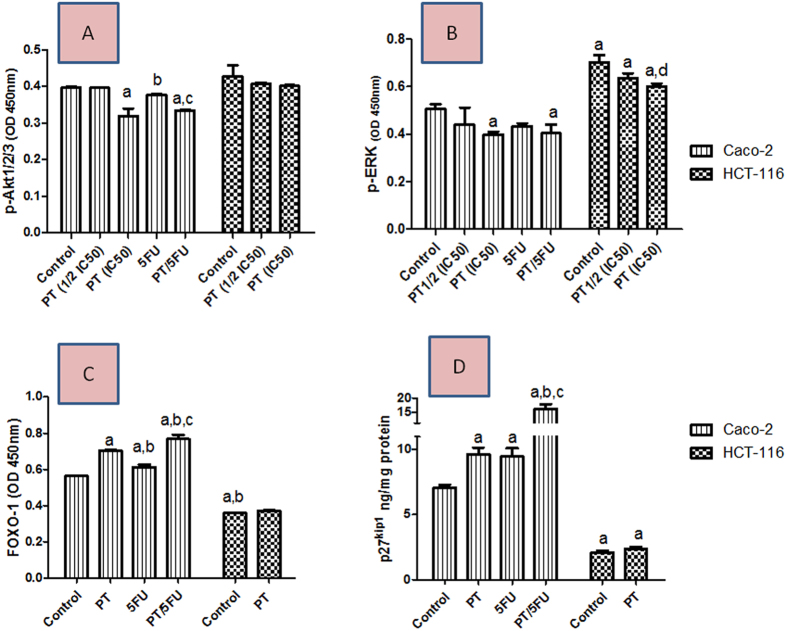
Effect of PT on Akt1/2/3 activation (**A**), ERK1/2 (**B**), FOXO1 (**C**) and p27^kip1^(D) expression in Caco-2 and HCT-116 colon cancer cell lines using ELISA assay. Data are means ± SD (n = 3). The experiment was done in triplicates. a indicates significant difference from Caco-2 control at p < 0.05. b indicates significant difference from Caco-2/PT group at p < 0.05. c indicates significant difference from Caco-2/5-FU group at p < 0.05. d indicates significant difference from HCT-116 control at p < 0.05.

Evaluation of Akt phophorylation in colon cancer cell lines indicated that treatment of Caco-2 cells with PT at its IC_50_ significantly reduced Akt phosphorylation/ activation by about 19.6% (P < 0.001) compared to untreated cells (Fig. 4A). Akt activation was reduced by 5% and 15.6% (P < 0.001) after treatment with either 5-FU-alone or PT/5-FU combination.

### Effect of PT on ERK phosphorylation/activation

ERK phophorylation in HCT-116 was 28% (P < 0.001) higher compared to Caco-2. The activation of ERK in Caco-2 was reduced by 20.9% (P < 0.05) and 14% after single treatment with PT or 5-FU at their IC_50_ and by 20% (P < 0.05) in cells treated with PT/5-FU combination. Treatment of HCT-116 with PT at its IC_50_ reduced ERK activation by 14% (P < 0.05) (Fig. 4B).

### Effect of PT on FOXO-1 protein expression

The basal levels of FOXO-1 were higher in Caco-2 by about 1.6 folds (P < 0.001) compared to HCT-116. Treatment of Caco-2 cells with PT boosted FOXO-1 expression by 25% (P < 0.001) compared to control group (Fig. 4C). Furthermore, while 5-FU enhanced FOXO-1 by 9% (P < 0.01), co-treatment with PT boosted FOXO-1 by 36% (P < 0.001) versus control. The increase in FOXO-1 was 27% higher in the presence of PT which is a modulator for ER-β.

### Effect of PT on cell cycle phases of colon cancer cells

**Figure 5:**
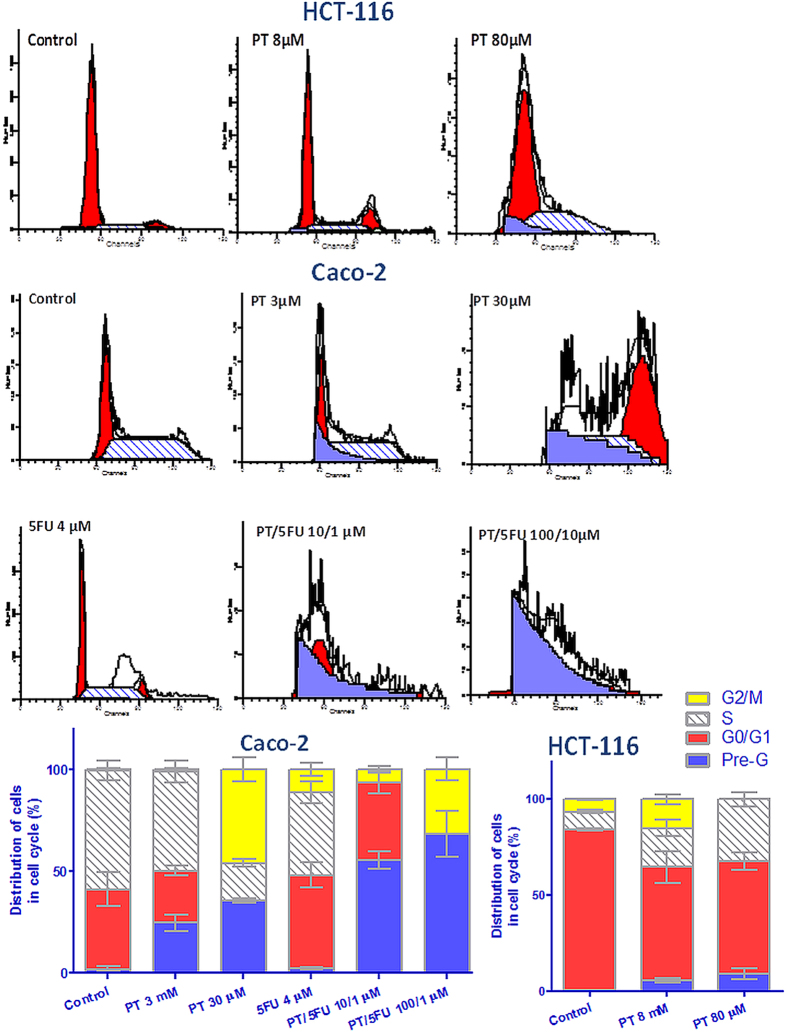
Effect of PT on DNA-ploidy flow cytometric analysis of Caco-2 and HCT-116 cells. The cells were treated with PT IC_50_ for 48 h. the experiment was done in triplicates. Data are means ± SD (n = 3).

Treatment of Caco-2 or HCT-116 cells with PT induced significant alterations in cell cycle phases (Fig. 5). Treatment of Caco-2 with low concentration of PT (3 μM) increased the accumulation of cells at pre-G phase by 12 fold (P < 0.05). Exposure of Caco-2 cells to 30 μM PT increased the percentage of cells at G2/M phase by 52% (P < 0.001) with concomitant increase in cells at pre-G phase by 17.5 fold (P < 0.001). Moreover, PT (30 μM) treatment led to reduction in the percentage of cells at G0/G1 and S-phases by 99% and 69% (P < 0.001), respectively compared to control. Treatment with PT/5-FU combination (10/1 μM) completely abolished cells at S-phase with concurrent increase in cells at pre-G by 27.5 fold (P < 0.001). High concentration of PT/5-FU (100/10 μM) produced complete abolishment of cells at G0/G1 and S-phases with concomitant increase in cells at G2/M by 42% (P < 0.001) as well as Pre-G phase by 34 fold (P < 0.001). Treatment of HCT-116 cells with 8 μM PT resulted in 2.2 folds (P < 0.05) arrest of cells at the S-phase coupled with 9.8 fold (P < 0.05) and 2.3 fold (P < 0.001) increase in cells at pre-G and G2/M phases. A subsequent reduction in percent of cells at G0/G1 by 29% (P < 0.01) was observed. It is noteworthy that cells at G2/M were eliminated by 80 μM PT treatment. Treatment with 80 μM PT also reduced the accumulation of cells at G0/G1 phase by 29.5% (P < 0.01). A significant increase in cells at S-phase and pre-G phase by 15.7(P < 0.01) and 3.5 (P < 0.001) folds was observed with the same treatment.

### Effect of PT on P27^kip1^ protein expression

Exposure of Caco-2 cells to PT significantly increased P27^kip1^ protein expression levels by 1.36 fold (P < 0.05) compared to control. The expression levels of P27^kip1^ were boosted by 1.34 fold (P < 0.05) and 2.28 fold (P < 0.001) after treatment with 5-FU alone or combined with PT. No change was observed in the levels of P27^kip1^ in HCT-116 cells (Fig. 4D).

### Effect of PT on Bax and Bcl-2 protein expression

**Figure 6:**
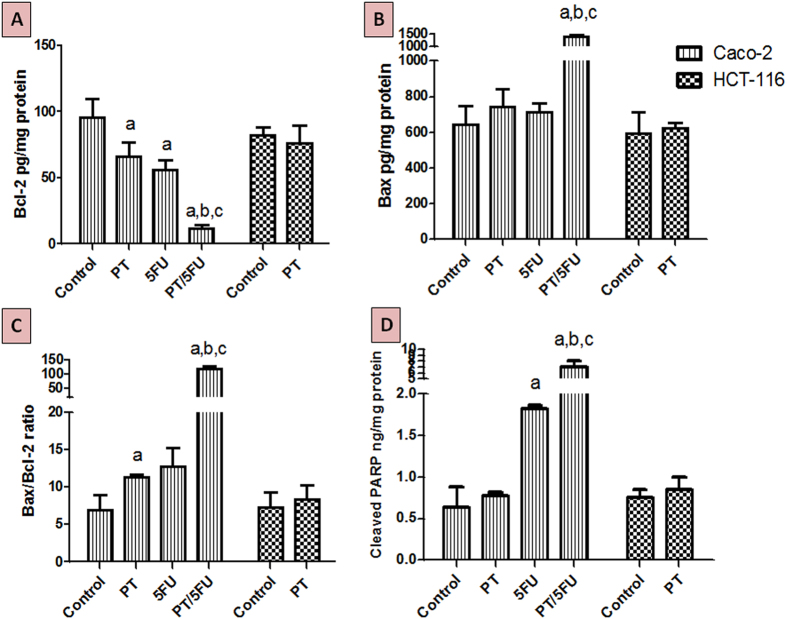
Effect of PT on the protein levels of (**A**) Bax, (**B**) Bcl-2, (**C**) Bax/Bcl-2 ratio and (**D**) PARP in Caco-2 and HCT-116 colon cancer cell lines using ELISA assay. Data are means ± SD (n = 3). The experiment was done in triplicates. a indicates significant difference from Caco-2 control at p < 0.05. b indicates significant difference from Caco-2/PT group at p < 0.05. c indicates significant difference from Caco-2/5-FU group at p < 0.05.

Treatment of Caco-2 cells with PT resulted in an increase in Bax level (pro-apoptotic) by about 15% together with a significant decrease in Bcl-2 (anti-apoptotic) level by 31% (P < 0.05) compared to untreated cells (Fig. 6A,B). Co-treatment with PT/5-FU increased Bax level by 2.14 fold (P < 0.001) and reduced Bcl-2 level by 87.7% (P < 0.001). Bax/Bcl-2 ratio was significantly boosted by 9.4 fold (P < 0.001) compared to 5-FU alone treatment. Likewise, treatment of HCT-116 with PT produced a slight increase in Bax level (5%) and decrease in Bcl-2 level (6%) compared to control. PT treatment resulted in increased Bax/Bcl-2 ratio by 14% in HCT-116 cells (Fig. 6A,B).

### Effect of PT on the level of cleaved PARP

Treatment of Caco-2 or HCT-116 cells with PT produced an increase in the levels of cleaved PARP by 1.22 and 1.1 fold, respectively compared to untreated controls (Fig. 6D). Treatment of Caco-2 with 5-FU alone or in combination with PT boosted PARP level by 2.9(P < 0.05) and 11 fold (P < 0.001). Combined treatment boosted the level of cleaved PARP by 3.8 fold (P < 0.001) compared to 5-FU alone.

## Discussion

In the current investigation, the cytotoxicity of PT alone and in combination with 5-FU was tested and compared in Caco-2 and HCT-116 colon cancer cells. ER-β is reported to be essential for PT beneficial effects in mammalian cells^19^. To test the hypothesis that PT cytotoxicity in colon cancer cells is mediated by ER-β, we assessed the cytotoxicity of PT in two colon cancer cell lines that exhibit differential ER-β expression. Previous reports indicated that ER-β is constitutively expressed in Caco-2 cells^20^ while HCT-116 cells have lower expression level of ER-β^21^,^22^ which was confirmed by our data. Cytotoxicity studies indicated that PT exhibited a more potent cytotoxic effect in Caco-2 compared to HCT-116 cells as indicated by its IC_50_ in both cells. These results are in line with the work of Nutakul *et al.*^18^ who indicated that PT is more potent in inhibiting the colony formation of Caco-2 compared to HCT-116. Additional support for the role of ER-β in this process stems from our data indicating that treatment of Caco-2 with PT boosted the expression of ER-β in these cells while only inducing a slight increase in ER-β expression in HCT-116. Treatment of both cell lines with 5-FU showed a concentration-dependent cytotoxicity, with Caco-2 being less sensitive to treatment than HCT-116 cells. Co-treatment with PT/5-FU was more effective in Caco-2 as indicated by Calcusyn^®^ Synergy analyses showing lower CI values and higher dose-reduction indices compared to HCT-116 cells. Treatment with 5-FU alone reduced ER-β expression by 20.5% (P < 0.001) which is consistent with the work of *Yu-Jing Fang et al.*^23^. Combined treatment with PT/5-FU improved 5-FU- mediated suppression of ER-β by 8.9%.

Testing the cytotoxicity of PT in normal non-cancer cells indicated that normal cells are more sensitive to its toxicity which is in line with PT previously reported antiproliferative effects in normal C2C12 myoblasts^19^. This finding indicates the need for a method of targeted delivery of PT to cancer cells to mitigate its potential toxic effects to normal cells.

Phosphatidylinositol-4,5-biphosphate 3-kinase (PI3K)/Akt pathway is known to play an important role in the pathogenesis and aggressiveness of many types of cancers including CRC^24^. Expression of ER-β was reported to repress PI3K/Akt signaling in glioma^25^, ovarian cancer^26^ and breast cancer cells^27^. Therefore, we tested the effect of PT on Akt phosphorylation in colon cancer cells. Treatment of Caco-2 with PT at a concentration equal to its IC_50_ significantly suppressed Akt phosphorylation, while there was no change in the Akt phosphorylation in HCT-116. Co-treatment of Caco-2 with PT/5-FU suppressed Akt activation by 10% greater than that observed with 5-FU-alone treatment. PT was previously reported to downregulate Akt signaling in COLO 205 colon cancer cells^17^. Previous reports indicated that ER-β expression downregulates ERK1/2 activation in breast cancer cells^28^. The tested cells Caco-2 and HCT-116 are known to be different in ERK1/2 activation status because Caco-2 is KRAS wild type while HCT-116 is KRAS mutant^29^. Therefore, ERK1/2 constitutive activation is higher in HCT-116 which was confirmed in the current work. Furthermore, PT treatment suppressed ERK1/2 activation in both cell lines with greater suppression of ERK1/2 phosporylation in Caco-2. This is in line with PT previously reported ability to suppress ERK1/2 activity in lung cancer cells^30^.

Forkhead-Box Class-O1 (FOXO-1) transcription factor is a downstream to ER-β that mediates ER-β pro-apoptotic functions^31^,^32^. Moreover, the activity of FOXO-1 is inhibited by Akt phosphorylation^33^,^34^. Our data indicate that the baseline level of FOXO-1 was higher in Caco-2 compared to HCT-116 cells. This could be partly explained by our data which indicated higher expression levels of ER-β in Caco-2. ER-β maintains FOXO-1 expression through tethering on *FOXO-1* promoter^31^. Treatment of Caco-2 with PT enhanced FOXO-1 expression compared to untreated cells which can be explained at least partly by PT effect on ER-β expression in Caco-2. This finding is supported by previous studies with prostate cancer cells, where CAPE treatment enhanced ER-β as well as FOXO-1 protein abundance in PC-3 cells^32^. While 5-FU enhanced FOXO-1 by 9% (P < 0.01), co-treatment with PT boosted FOXO-1 by 36% (P < 0.001) versus control. Treatment with 5-FU was previously reported to enhance FOXO-1 levels in breast cancer cells^35^. The increase in FOXO-1 in our study was 27% higher in the presence of PT which is an ER-β modulator. This gains support by our molecular docking studies which revealed that PT exhibits strong interaction with the receptor active site that is comparable to its ligand 17-β estradiol.

Upregulation of FOXO-1 signaling is associated with induction of cell cycle arrest and apoptosis^36^. We therefore evaluated the effect of PT on those cellular processes. Cell cycle analysis indicated a significant G2/M arrest in Caco-2 coupled with an increase in the percentage of cells in pre-G phase, which is indicative of apoptosis, after treatment of cells with PT IC_50_. These findings are suggestive of the induction of mitotic catastrophe^37^. Co-treatment of Caco-2 with PT/5-FU (10/1 μM) increased the percent of cells at pre-G. Combined treatment at a higher concentration of PT/5-FU (100/10 μM) resulted in a complete abolishment for the cells at G0/G1 or S-phases with significant increase in the percent of cells at G2/M or pre-G phases. Reduction of the percent of dormant cell population at G0 phase by increasing the percent of cells in S- or G2/M phases was previously shown to sensitize Caco-2 to 5-FU cytotoxicity^38^. Therefore, the increased abundance of cells at G2/M together with suppression of cells at G0/G1 by PT can also justify its chemosensitizing effect. Although pre-G phase was also elevated after treatment of HCT-116 with PT, the main cell cycle arrest was at S-phase. PT-induced arrest at S-phase was previously reported in bladder cancer^39^ and leukemia cells^40^. The increased number of cells at pre-G after PT treatment was also reported in COLO 205 colon cancer cells^17^.

Reduced level of p27^kip1^, a cyclin-dependent kinase inhibitor, is linked to poor prognosis and elevated rate of tumor relapse in patients with CRC^41^,^42^. We therefore evaluated the effect of PT on its level. Treatment of Caco-2 but not HCT-116 with PT resulted in a significant upregulation of p27^kip1^ protein. Co-treatment of Caco-2 with PT/5-FU significantly boosted p27^kip1^expression. This finding further support the data generated from cell cycle analysis. It is noteworthy that p27^kip1^ is a transcriptional target for FOXO-1^43^. Therefore, PT-mediated increase in p27^kip1^expression in Caco-2 can be attributed to its effects on FOXO-1.

The proapoptotic effects of PT, evidenced by increased accumulation of cells at pre-G phase, were further investigated. Both Bax and Bcl-2 proteins are key regulators for the intrinsic apoptosis pathway. The balance between the proapoptotic protein Bax and the antiapoptotic protein Bcl-2 is a critical factor for the cells’ threshold to undergo apoptosis^44^,^45^. This ratio is also an important determinant for the sensitivity of cancer cells to chemotherapy including 5-FU^46^. Previous studies indicated that PT downregulated Bcl-2^39^,^47^,^48^ and upregulated Bax^48^,^49^ in several cancer cell types. In the current study, PT treatment significantly increased Bax/Bcl-2 ratio in Caco-2 compared to control. Combined treatment of Caco-2 with PT/5-FU increased Bax/Bcl-2 ratio by 17 fold compared to control and 10 fold compared to 5-FU-alone treatment. On the other hand, HCT-116 treatment with PT led only to a slight increase in the ratio. These findings support our DNA-ploidy data indicating that the pro-apoptotic effects of PT were more evident in Caco-2 cells. Additionally, the percent of Caco-2 cells at pre-G after combined treatment was significantly higher compared to single treatments of either PT or 5-FU. ER-β expression in colon cancer cells^21^,^50^ and other tumor types^51^,^52^ is directly correlated with apoptosis. Our data for PT effects in Caco-2 versus HCT-116 cells, are concordant with these observations. Activation of Akt leads to tumor resistance to apoptosis^53^. The significant enhancement of Bax/Bcl-2 ratio in PT alone and combined treatments can be linked to the effect of PT on Akt activation in Caco-2 cells. Since, treatment with PT inhibited Akt activation in Caco-2 only and combined PT/5-FU treatment inhibited Akt activation to a greater extent compared to 5-FU-alone treatment. Poly (ADP-ribose) polymerase (PARP) is a crucial enzyme for DNA repair. This enzyme is inactivated (cleaved) via caspase-3 at the end of apoptosis cascade leading to accumulation of unrepaired DNA with subsequent cell death^54^. Treatment of cells with PT increased cleaved PARP levels in Caco-2 to a greater extent compared to HCT-116. Combined treatment significantly increased cleaved PARP levels in Caco-2 compared to 5-FU-alone. This is concordant with the observed effects of PT on the apoptosis markers (Bax/Bcl-2 ratio and the percent of cells at the pre-G phase) in Caco-2 cells.

In summary, it could be concluded from these studies that, PT synergizes the cytotoxic effects of 5-FU in Caco-2 colon cancer cells. PT exhibited higher potency in Caco-2 cells, which express higher levels of ER-β, compared to HCT-116. These results provide a rationale for novel combination treatment strategies, especially for patients with 5-FU-resistant tumors expressing ER-β protein.

## Materials and Methods

### Chemicals

Pterostilbene (PT), sulforhodamine-B (SRB) and dimethyl sulfoxide (DMSO) were purchased from Sigma-Aldrich (St. Louis, MO). RPMI-1640 medium, fetal bovine serum and other cell culture materials were purchased from Lonza (Basel, Switzerland).

### Cell culture

The human colon cancer cell lines Caco-2 and HCT-116 were obtained from the National Cancer Institute (Cairo, Egypt). Cells were maintained in RPMI-1640 medium supplemented with 100 μg/mL of streptomycin, 100 units/mL of penicillin and 10% of heat-inactivated fetal bovine serum in a humidified 5% (v/v) CO_2_ atmosphere at 37 °C.

### Cytotoxicity Assay and Synergy Analysis

PT was dissolved in DMSO and kept at a stock concentration of 100 mM. Initially, single-drug concentration– effect curves of PT and 5-FU were assessed. Seeding was done at a density of 3,000 cells/well in 96-well plates. Cells were exposed to different treatments for 72 h during which 5 different drug concentrations (0–10^3^ μM) were tested. Cytotoxicity was assessed at the end of drug exposure using the sulforhodamine-B (SRB) assay as described previously^55^. The optical density was measured at 545 nm using a microplate reader (ChroMate- 4300, FL, USA). Results were expressed as the relative percentage of absorbance compared to control. Experimental conditions were tested using three replicates (three wells of the 96-well plate per experimental condition) and all experiments were performed in triplicates. Control wells were exposed to the same concentration of DMSO (1%) used in the highest concentration of PT series. Cell viability was >99% compared to untreated cells. Half-maximal inhibitory concentration (IC_50_), the drug concentration at which 50% growth inhibition is achieved, was calculated according to the equation for Boltzman sigmoidal concentration–response curve using the nonlinear regression fitting models (Graph Pad, Prism Version 5). Drug interactions were analyzed by CalcuSyn program, Version 2.1 (Biosoft, Cambridge, UK) based on the analytical method of Chou and Talalay^56^. The analytical method of Chou and Talalay^57^ yields two parameters that describe the interactions in a given combination: the combination index (CI) and the dose reduction index (DRI). The combination index (CI) equation is based on the multiple drug-effect equation of Chou–Talalay derived from enzyme kinetic models, where CI of <1 indicates synergism, CI of 1 or close to 1 indicates additive effects, and CI of >1 indicates antagonism. The DRI denotes the factor by which the dose of each drug in a combination may be reduced at a given effect level compared with the dose when each drug is used alone. As such, the DRI is important in clinical situations, where dose reduction can lead to reduced toxicity while retaining the therapeutic efficacy. DRI >1 is beneficial, and the greater the DRI value, the higher the dose reduction is for a given therapeutic effect.

### Cytotoxicity testing in normal cells

Normal placental trophoblasts were utilized to assess the selective cytotoxicity of PT. The villous 3A cytotrophoblast first trimester placental cell line (CRL-1584, ATCC, Manassas, VA) was used. The cells were cultured in EMEM medium supplemented with 10% fetal bovine serum and 1% penicillin/streptomycin in a humidified incubator at 37 °C supplemented with 5% CO_2_. The cells were seeded at density of 75 × 10^3^ cells/well in 24-well plates and incubated overnight to allow for optimum attachment. The following day, cells were exposed to PT at a concentration close to its IC_50_ observed in Caco-2 and HCT-116 cancer cells (40 and 80 μM) and the incubation was continued for 72 h. At the end of the exposure period the cells were stained using SRB as described previously^55^. Results were expressed as the relative percentage of optical density at 545 nm compared to control.

### Molecular Docking

Docking of PT was performed using Discovery Studio 4/CDOCKER protocol (Accelrys Software Inc.). Protein crystallographic structures of human ER-β active site (PDB code 1×7R) were obtained from Protein Data Bank (PDB; http://www.rcsb.org/pdb). The proteins were prepared for docking process according to the standard protein preparation procedure integrated in Accelry’s Discovery Studio 4. The 3D structures of 17-β estradiol and PT were drawn and refined using CHARMM force field with full potential. Docking study of PT was perfumed on ER-β active site. All the docking simulations were run using CDOCKER protocol. The binding energy was calculated as a score to rank the docking poses. Ten docking poses were calculated for each ligand. Docking poses were ranked according to their –CDOCKER interaction energy, and the top poses were chosen for analysis of interactions.

### Assessment of ER-β and FOXO-1 expression

The protein abundance ER-β in both Caco-2 and HCT-116 was assessed using immunocyotochemistry as previously described by Tolba *et al.*^32^, after culturing the cells on ibidi^®^ μ-Chamber 12-well slides (Munich, Germany). ELISA kit for ER-β (AMS Biotechnology Ltd, UK) was used to determine the expression level of ER-β in cell lysates exposed or unexposed to PT treatment. Total FOxO-1 Cell-Based Colorimetric ELISA Kit (Ameritech Biomedicines, Houston, Tx) was used to determine the levels of FOXO-1 in the fixed cells according to the manufacturer’s instructions. The cells were treated with PT at a concentration equal to its IC_50_ (30 μM for Caco-2 and 80 μM for HCT-118) for 48 h. Caco-2 cells were treated with 5-FU 40 μM and PT/5-FU combination 10/1 μM.

### Assessment of Akt and ERK activation/phosphorylation

The effect of PT on AKT activation in the tested colon cancer cell lines was assessed using phospho-AKT 1/2/3 (Ser473) and phospho-ERK1/2 InstantOne™ ELISA kit (eBiosciences, San Diego, CA). This kits were developed to detect phosphorylation at AKT1/2/3(Ser473) and ERK1/2(Thr202/Tyr204, Thr185/Tyr187) respectively. Caco-2 and HCT-116 cells were seeded into 96- well plates at a density of 3 × 10^4^ cells/well and incubated overnight to allow for attachment. PT was added at concentrations equal to the IC_50_ and half of the IC_50_ value for each cell line. After 48 h of exposure, the medium was discarded and the wells were washed twice with Hank’s buffered salt solution. Akt activity was determined in the cell lysates as previously described by Tolba *et al.*^32^.

### DNA-flow cytometry

Caco-2 or HCT-116 cells at a density of 3 × 10^5^ cells were exposed to different treatments for 48 h. Treatments of Caco-2 were done at 3, 30 μM PT, 4 μM 5-FU, 10/1 and 100/10 μM of PT/5-FU. Treatments of HCT-116 were done using 8 and 80 μM PT. The cells were collected by trypsinization, washed in PBS and then fixed in ice-cold absolute alcohol. The cells were then stained using CycleTEST^TM^ PLUS DNA Reagent Kit (BD Biosciences, San Jose, CA) according to the manufacturer’s instructions. Cell-cycle distribution was determined using a FACSCalibur flow cytometer (BD Biosciences, San Jose, CA).

### Assessment of p27^kip1^ and apoptosis markers

The levels of p27^kip1^, Bax, Bcl-2 and cleaved PARP in the cell lysates collected at 48 h exposure were detected using specific ELISA kits. Human p27kip1 simple step ELISA kit was purchased from Abcam (Cambridge, MA), while kits for Bax and Bcl-2 were obtained from Abnova (Taiwan). ELISA kit for cleaved PARP was purchased from Invitrogen (Camarillo, CA). All the assay procedures were conducted according to the manufacturer’s recommendations.

### Statistical Analysis

Data are presented as means ± SD. Individual groups were compared using the two-tailed independent Student’s t-test. Multiple group comparisons were carried out using one-way analysis of variance (ANOVA) followed by the Tukey-Kramer test for post-hoc analysis. Statistical significance was accepted at a level of P < 0.05. All statistical analyses were performed using GraphPad InStat software, version 3.05 (GraphPad Software, Inc. La Jolla, CA, USA). Graphs were sketched using GraphPad Prism software, version 5.00 (GraphPad Software, Inc. La Jolla, CA, USA).

## Additional Information

**How to cite this article**: Tolba, M. F. and Abdel-Rahman, S. Z. Pterostilbine, an active component of blueberries, sensitizes colon cancer cells to 5-fluorouracil cytotoxicity. *Sci. Rep.* **5**, 15239; doi: 10.1038/srep15239 (2015).

## Supplementary Material

Supplementary Information

## Figures and Tables

**Table 1:** Synergy analysis for PT/5-FU combination.

PT/5-FU combination			Caco-2 cellline	HCT-116 cellline
PT μM	5-FUμM	Fa†	CI*	CI
1	0.1	0.05	0.505	6.1e + 005
10	1	0.3	0.605	537.871
100	10	0.7	0.702	1.838
1000	100	0.95	0.846	0.002

^†^Fa, fraction affected; ^*^CI, combination index. CI of <1 indicates synergism, CI of 1 or close to 1 indicates additive effects, and CI of >1 indicates antagonism.

**Table 2:** Dose reduction index (DRI)† for PT/5-FU combination.

PT/5-FU combination		
Fa	DRI (Caco-2 cell line)	DRI (HCT-116 cell line)
0.05	42.1	1.64e-006
0.3	46.48	0.002
0.5	48.86	0.032
0.7	50.39	0.254
0.95	55.695	619.58

^†^DRI denotes the factor by which the dose of each drug in a combination may be reduced at a given effect level compared with the dose when each drug is used alone. DRI >1 is considered beneficial. The greater the DRI value, the higher the dose reduction is for a given therapeutic effect.
