# Supplementary material for: Pterostilbine, an active component of blueberries, sensitizes colon cancer cells to 5-fluorouracil cytotoxicity
Source: Sci Rep. 2015 Oct 16;5:15239. doi: 10.1038/srep15239 (PMC4608003; doi:10.1038/srep15239)
Supplement: Supplementary Information [file srep15239-s1.pdf]

Pterostilbine, an active component of blueberries sensitizes colon cancer cells to 5-fluorouracil cytotoxicity

Mai F. Tolba[a,b], Sherif Z. Abdel-Rahman[c,*]

[a] Department of Pharmacology and Toxicology, Faculty of Pharmacy, Ain Shams University, Cairo 11566, Egypt, tolba.mf@pharma.asu.edu.eg ; [b] Biology Department, School of Sciences and Engineering, The American University in Cairo, New Cairo, Egypt; [c]Department of Obstetrics and Gynecology, The University of Texas, Medical Branch, Galveston 77555, Texas, sabdelra@utmb.edu

……………………………………………………………………………………………………

Running title: Pterostilbine sensitizes colon cancer cells to chemotherapy

***Corresponding author:**

Sherif Z. Abdel-Rahman, Ph.D.

Associate Professor

Department of Obstetrics and Gynecology

The University of Texas Medical Branch

7.138 Medical Research Building

Galveston, TX 77555-1066, USA

Tel: (409) 772-9111

Fax: (409) 772-2261

Email: sabdelra@utmb.edu

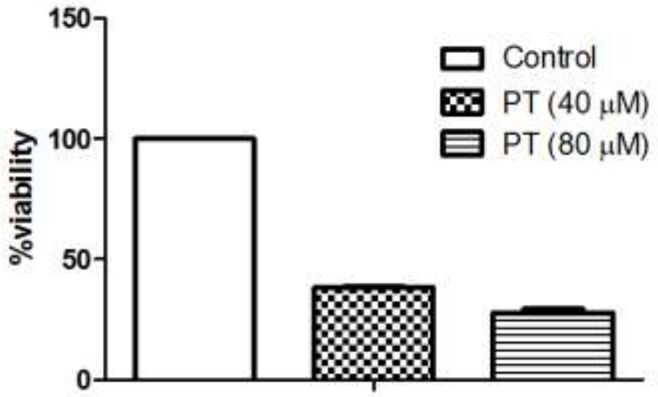

Supplementary Fig 1 Effect of PT on the viability of normal placental cytotrophoblasts (CRL-1584, ATCC, Manassas, VA). Data are means ± SD (n=3).
